# Clinicopathological characteristics, survival outcomes and prognostic factors in pleomorphic carcinoma: a SEER population-based study

**DOI:** 10.1186/s12890-022-01915-1

**Published:** 2022-03-31

**Authors:** Zhongzhong Chen, Jiachang Liu, Lingfeng Min

**Affiliations:** 1 Department of Respiratory and Critical Care Medicine, First Affiliated Hospital of Anhui University of Science and Technology (Huainan First People’s Hospital), Huainan, 232007 Anhui China; 2 Department of Respiratory and Critical Care Medicine, Northern Jiangsu People’s Hospital, Dalian Medical University, Clinical Medical College of Yangzhou University, Yangzhou, 225001 Jiangsu China

**Keywords:** Clinicopathological characteristics, Pleomorphic carcinoma, SEER database, Treatment and outcome

## Abstract

**Background:**

Pulmonary pleomorphic carcinoma (PPC) is a rare tumor, and it usually has an aggressive clinical course and poor prognosis. We aim to analyze the clinicopathological features, management and prognostic factors of pulmonary pleomorphic carcinoma.

**Patients and methods:**

Using the surveillance, epidemiology, and end results (SEER) database, we identified 461 patients of pulmonary pleomorphic carcinoma from 2004 to 2014 including clinicopathological characteristics, treatment modalities and outcome data.

**Results:**

The mean age of all PPC patients was 66 years and 58% of the patients were male. Most patients (80%) were white people, 53% were found in the right lung, and lesions were mostly observed in upper lobe (56%). The median overall survival was 9 months and overall 1-, 3- and 5-year survival rate was 45%, 29%, 23%. In Kaplan–Meier analysis, age, marital status, tumor primary site, gender, laterality, SEER summary stage, chemotherapy and surgery were associated with overall survival. Patients received surgery or chemotherapy had a better OS for patients with PPC. Multivariate Cox analysis revealed that SEER summary stage, age, surgery and chemotherapy were found to be independently associated with the OS. Surgery could significantly prolong survival in patients with localized stage and regional stage (HR = 0.120, 95% CI 0.038–0.383, *p* < 0.001; HR = 0.351, 95% CI 0.212–0.582, *p* < 0.001) while it did not have great impact on survival in patients with distant stage (*p* = 0.192). Chemotherapy decreased risk of death by 46% (HR = 0.544, 95% CI 0.393–0.752, *p* < 0.001) for patients with distant stage, whereas chemotherapy did not confer survival benefits to patients with localized stage and regional stage. But radiation did not have great impact on survival of patients with different stages in this study.

**Conclusions:**

PPC mostly occurred in white people, with a median age of 66 years, and men were more susceptible to this disease. The SEER summary stage, age, surgery and chemotherapy were independently associated with prognosis. Surgery should be considered for the PPC patients with localized stage or regional stage, and chemotherapy should be recommended for the treatment of patients with distant stage.

## Background

Pleomorphic carcinoma (PC) is a rare type of poorly differentiated non-small cell lung carcinoma, containing at least a 10% component of spindle and/or giant cells, or a carcinoma that consists entirely of spindle and/or neoplastic giant cells [1, 2]. According to 2004 WHO Classification of Tumours Editorial Board, PC was one of the five subtypes of sarcomatoid carcinomas, including spindle cell carcinoma, giant cell carcinoma, carcinosarcoma, pleomorphic carcinoma, and pulmonary blastoma [3]. But according to the 2021 WHO Classification [4], PC is a subtype of sarcomatoid carcinoma, while PC has three subtypes: pleomorphic carcinoma, giant cell carcinoma and spindle cell carcinoma. PC could be found in almost any site in the body and most of this originated from the respiratory system. This tumor originating from the respiratory system is called pulmonary pleomorphic carcinoma (PPC). The clinical symptoms of PC are non-specific compared with other non-small cell lung cancers but the prognosis is relatively poor [5, 6]. In the past, because of the lack of a conclusive definition and the rarity of pulmonary pleomorphic carcinoma, no consensus had been reached regarding its diagnosis [7]. After 1999, a unified diagnostic standard was established for PPC, which was recognized as a neoplasm with pleomorphic, sarcomatoid or sarcomatous elements in the category of carcinomas [7]. Because of pleomorphic differentiation and atypical cell morphology, the definitive diagnosis of pleomorphic carcinoma may be rendered only in surgical specimens [9].

PPC had a worse outcome than other NSCLC and the optimal treatment of patients remains unknown [10]. Treatment modalities of PPC has varied across previous reports. In the early stage of this type of cancer, surgical excision may be the preferred method of treatment and the key to prevent recurrence and metastasis [11]. However, some researchers documented that the prognosis of patients with pleomorphic carcinoma was poor despite surgery and adjuvant chemotherapy, even in the case of local disease [12]. Effective therapeutic modalities combined with surgery should be evaluated to improve outcomes, even when the tumor is at an early stage [11, 13, 14]. Thus, systemic therapy of PPC needed to be explored.

As PPC is a rare histologic subtype, patients with this type of pathology are rare [15]. A review of the literature did not find any specific systematic reports of this tumor [16]. The general population, clinicopathological characteristics of patients with this type of pathology are not well known. Therefore, we conducted a retrospective analysis of patients with PPC by using the surveillance, epidemiology, and end results (SEER) database, to predict the trends in overall demographic features, basic clinicopathologic characteristics and compare treatment modalities and outcome of PPC.

## Patients and methods

### Patients

The SEER database was used to extract basic clinicopathologic characteristics and survival data. Clinicopathological information was extracted by using the SEER*Stat 8.5.0 software. According to the International Classification of Diseases for Oncology codes (ICD-O-3), PC cases were identified by histology codes 8022/3 (pleomorphic carcinoma). Preliminary selection criteria for study cases included: (1) diagnosis of PC; (2) histological confirmation of pleomorphic carcinoma (8022/3); (3) diagnosis between 2004 and 2014. Exclusion criteria were: (1) not the first primary malignancy; (2) unknown SEER summary stage; (3) unknown survival time; (4) unknown treatment.

SEER does not uncover sensitive patient information, and we registered the study with the IRB and received a clearance.

### Statistical analysis

In the present study, we used overall survival (OS) as the time from diagnosis to death from any cause, and patients alive were censored at the time of the last recording. Patients who died from other causes unrelated to pleomorphic carcinoma diagnosis or were alive were censored on the date of death or the date of last contact.

For statistical analysis, the demographic and clinicopathologic parameters were selected on the following propensity factors: age, gender, race, marital status, tumor primary site, treatment modality, outcome status. We identified some of all the cases don’t contain all these data. Statistical analysis was performed using the software Graph Pad Prism 7.0 and SPSS (version 22; IBM, Chicago, IL). Continuous data were compared using a Student’s t-test, and categorical data were compared using a Chi-square test. The OS was estimated using the Kaplan–Meier product-limit method and compared by log-rank test. To predict the predictors of the prognosis, the Cox proportional hazards model was used. In statistical analysis, variables with *p* < 0.1 in univariate analysis were included in a multivariate analysis. Univariate and multivariate Cox proportional hazard models were applied to identify factors associated with survival, with hazard ratios (HRs) and 95% confidence intervals (CIs) reported. The values of *p* < 0.05 were considered statistically significant, and all statistical tests were two sided.

## Results

### Patient characteristics

**Fig. 1:**
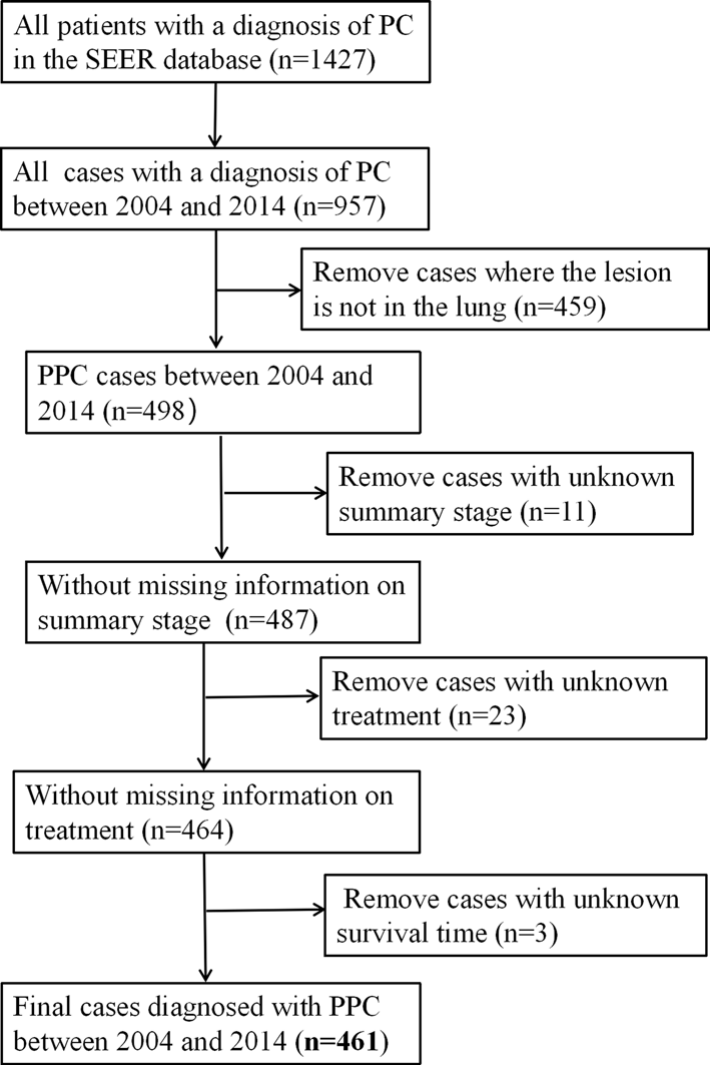
Flow diagram of the data selection process

**Fig. 2:**
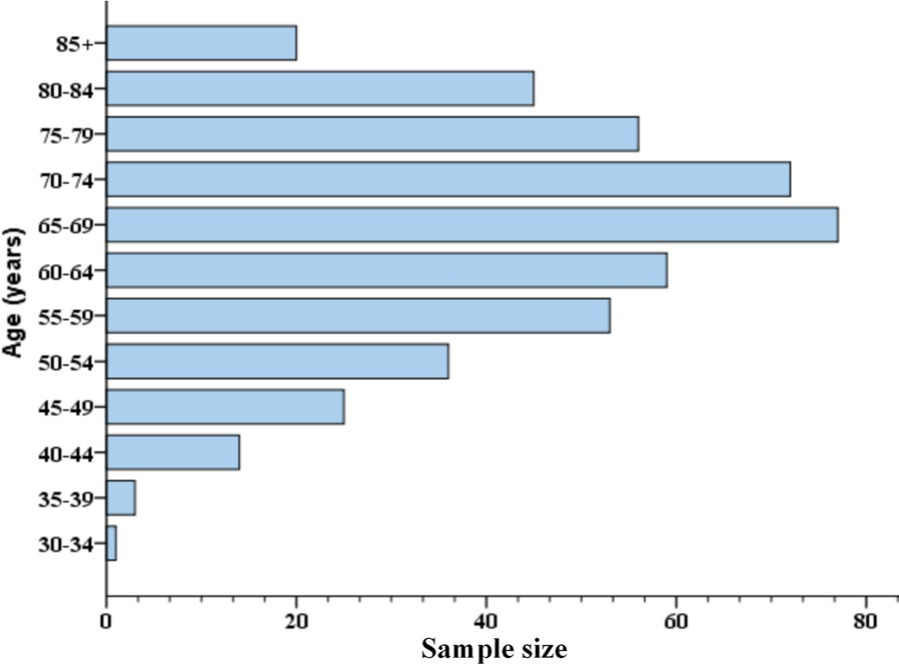
The distribution of age of all PPC cases registered in the SEER database

As shown in Fig. 1, of 1427 patients with a diagnosis of PC were included in the SEER database, we found 957 cases diagnosed with PC between 2004 and 2014. PC was found in almost any site of the body. However, the most common position of the PC was the lung and bronchus. This tumor originating from lung and bronchus is called pulmonary pleomorphic carcinoma (PPC). According to the above inclusion and exclusion criteria, 461 PPC patients were included from the SEER database. A peak incidence occurred at 65–75 years. Details of these analysis were shown in Fig. 2.

As described in Table 1, the PPC 461 patients had a median age of 66 (32–94), and most patients (51%) were married. Of the patients, 269 patients were male (58%) and 192 female (42%). Most patients (80%) were white people and 53% of lesions were observed in the right lung. Most lesions were observed in upper lobe (56%) and lower lobe (22%). Regarding treatment, patients who underwent chemotherapy or radiotherapy for the primary tumor trended to the younger age group. There was no significant difference in terms of tumor site or summary stage among the patients with PPC located at different age groups. There was a significant difference in marital status, gender or race among patients, which was expected according to the different age groups studied.

**Table 1:** Characteristics of 461 patients with PPC

Variable	Value (461)			
	≤ 66 years	> 66 years	Total number	*p*
*Gender*				**0.038**
male	144 (63%)	125 (54%)	269 (58%)	
Female	84 (37%)	108 (46%)	192 (42%)	
*Marital status*				**0.000**
Married	116 (51%)	119 (51%)	235 (51%)	
Single (unmarried)	46 (20%)	13 (6%)	59 (13%)	
Separated/divorced/widowed	66 (29%)	101 (43%)	167 (36%)	
*Race*				**0.000**
White	164 (72%)	206 (88%)	370 (80%)	
Black	51 (22%)	13 (6%)	64 (14%)	
Other	13 (6%)	14 (6%)	27 (6%)	
*Laterality*				0.315
Left	88 (39%)	106 (45%)	194 (42%)	
Right	128 (56%)	115 (49%)	243 (53%)	
Bilateral	12 (5%)	12 (5%)	24 (5%)	
*Primary site*				0.233
Upper lobe, lung	135 (59%)	124 (53%)	259 (56%)	
Lower lobe, lung	43 (19%)	56 (24%)	99 (22%)	
Main bronchus	7 (3%)	2 (1%)	9 (2%)	
Middle lobe, lung	10 (4%)	12 (5%)	22 (5%)	
Overlapping lesion of lung	33 (14%)	39 (17%)	72 (16%)	
*Summary stage*				0.530
Localized	35 (15%)	45 (19%)	80 (17%)	
Regional	86 (38%)	83 (36%)	169 (37%)	
Distant	107(47%)	105 (45%)	212 (46%)	
*Surgery*				0.742
Yes	117 (51%)	116 (50%)	233 (51%)	
No	111 (49%)	117 (50%)	228 (59%)	
*Radiation*				**0.001**
Yes	95 (42%)	63 (27%)	158 (34%)	
No	133 (58%)	170 (73%)	303 (66%)	
*Chemotherapy*				< **0.001**
Yes	114 (50%)	62 (27%)	176 (38%)	
No	114 (50%)	171(73%)	285 (62%)	

Statistical analyses were performed using SPSS

Bold means statistically significant value *p* < 0.05

### Patient survival

**Fig. 3:**
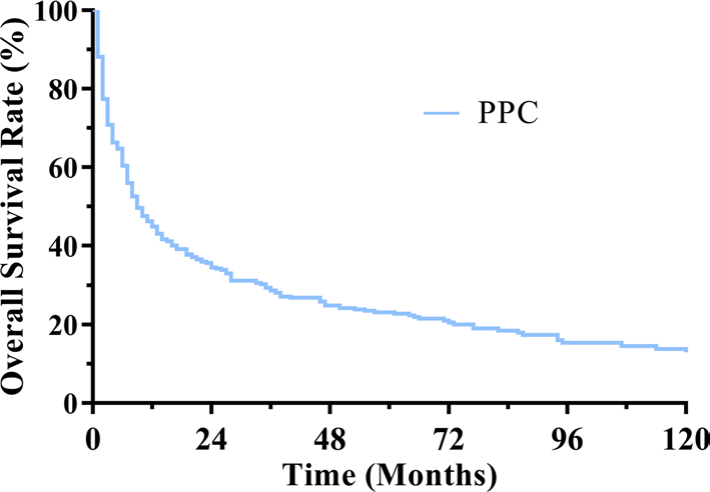
OS for patients with PPC

**Fig. 4:**
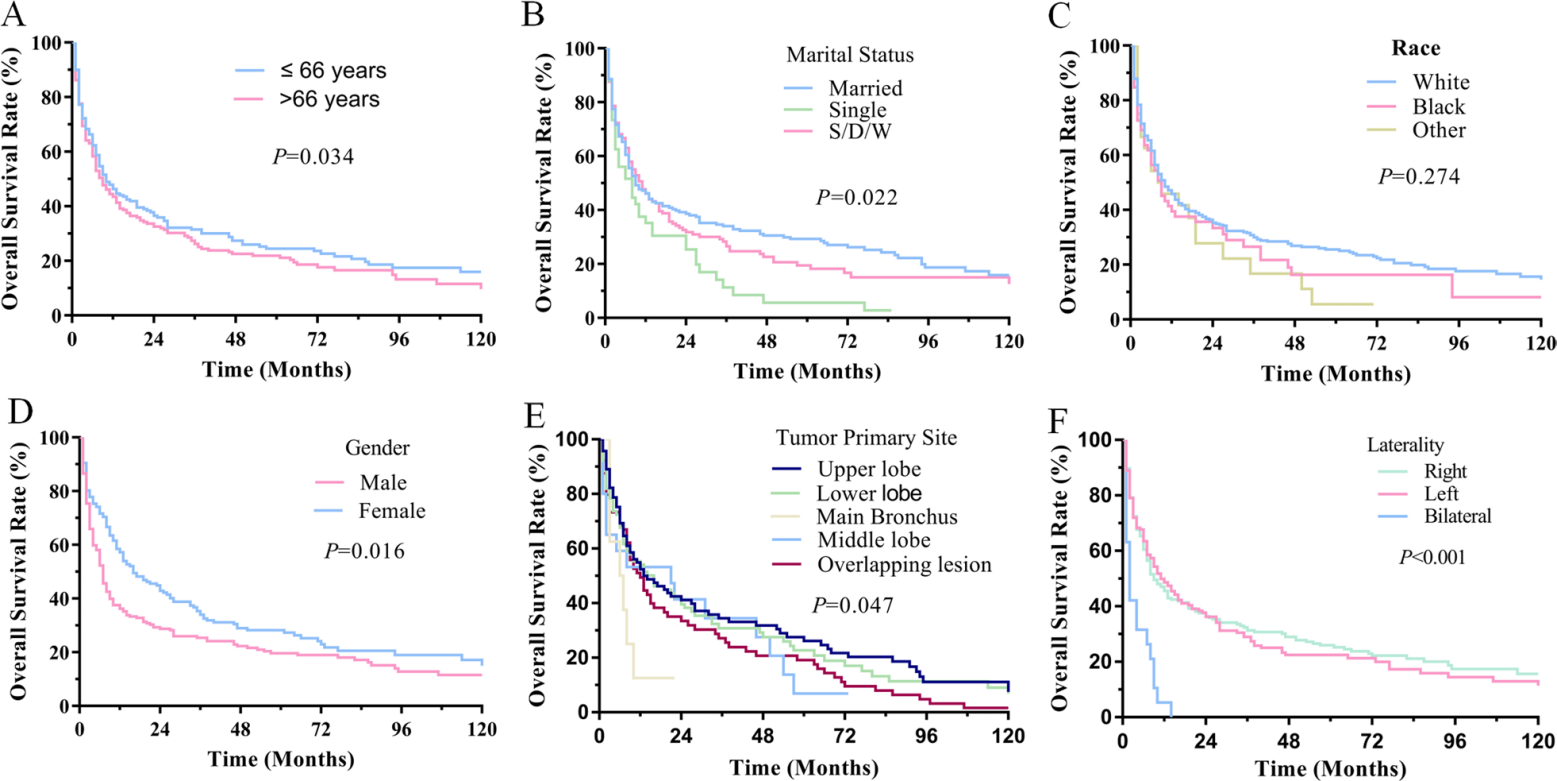
OS curves of cases with PPC compared according to **a** age; **b** marital status; **c** race; **d** gender; **e** tumor primary site; **f** laterality; Log-rank test was utilized to compare curves, and significance is presented on each pane

**Fig. 5:**
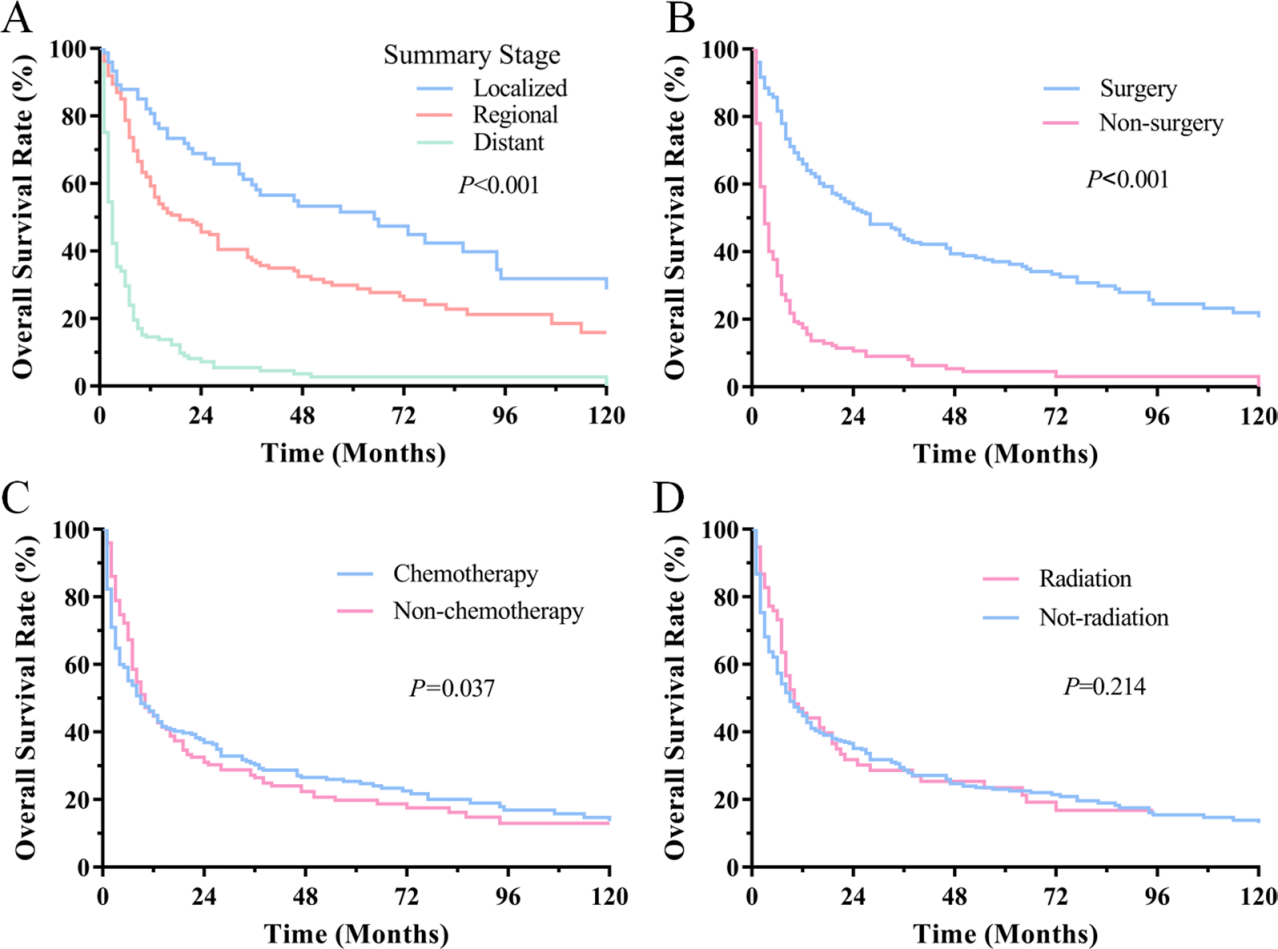
Survival curves of OS for patients with summary stage (**a**), surgery (**b**), chemotherapy (**c**), and radiotherapy (**d**); Log-rank test was utilized to compare curves, and significance is presented on each pane

Among the 461 PPC cases, the median OS was 9 months, the 1-, 3- and 5-year OS rate of PPC was 45%, 29%, 23%, respectively (Fig. 3). The Kaplan–Meier log-rank test indicated that age, marital status, tumor primary site, gender, laterality, SEER summary stage, chemotherapy and surgery were associated with OS. The younger (≤ 66 years) patients had better OS than older (> 66 years) and male had poorer OS than female (Fig. 4a, d). The patients had significantly different OS rates in terms of tumor primary sites while upper lobe had significantly higher OS than other sites (Fig. 4e). Patients with lesions in right lung tended to have better prognosis than those with left or bilateral lung (*p* < 0.001, Fig. 4f). Likewise, the marital status also affected the OS while race did not have great impact on survival (Fig. 4b, c). Besides, the patients with distant stages had significantly poorer OS than those with localized or regional stage (*p* < 0.001, Fig. 5a). To further figure out the clinical variables associated with survival, univariate and multivariate Cox proportional were applied to analysis. In univariate analysis, factors were proved to be significantly associated with OS including SEER summary stage, laterality, age, marital status, tumor primary site, chemotherapy and surgery. Patients with lesions in lung lobes had a better OS compared with those in the main bronchus (*p* < 0.001), but prognosis did not differ in patients with different lung lobes. Multivariate Cox analysis found that the > 66 years [*p* = 0.021, HR 95% CI 1.300 (1.040–1.624); ≤ 66 years—as Ref], regional stage [*p* = 0.002, HR 95% Cl 1.764 (1.239–2.514), localized stage—as Ref], distant stage [HR 95% Cl 4.223 (2.755–6.473), *p* < 0.001, localized stage—as Ref], surgery [*p* < 0.001, HR 95% CI 0.470 (0.339–0.653); no surgery—as Ref], chemotherapy [*p* = 0.002, HR 95% CI 0.683 (0.535–0.873); no chemotherapy—as Ref] were found to be independently associated with the OS.

### Patient treatment

Among these 461 patients, 51% (n = 233) patients received surgery, 33% (n = 176) patients received chemotherapy and radiotherapy was performed for 34% (n = 158). In our study, Kaplan–Meier curves revealed that surgery and chemotherapy were associated with improved survival, but we found that survival was not associated with having received radiation or not (Fig. 5). Patients received surgical resection (Fig. 5b) had significantly better prognosis than those who had not received surgery and the median OS improved from 2 to 28 months (*p* < 0.001). Likewise, patients received chemotherapy had better OS rates than those who had not received treatment (Fig. 5c). But we found that patients who received radiation could not improve survival than those who did not (Fig. 5d *p* = 0.214). After adjusting for the associated factors, multivariate Cox analysis determined that surgery and chemotherapy significantly predicted decreased mortality while the prognostic value of radiation was not significance (Table 2).

**Table 2:** Univariate and multivariate Cox proportional hazard analyses of clinical characteristics for overall survival rates in patients with PPC

Characteristic	Univariate		Multivariate	
	HR (95% CI)	*p* value	HR (95% CI)	*p* value
*Age*		**0.042**		**0.021**
≤ 66 years	Reference		Reference	
> 66 years	1.237 (1.008–1.519)	0.042	1.300 (1.040–1.624)	0.021
*Gender*		**0.020**		0.061
Male	Reference		Reference	
Female	0.781 (0.633–0.962)	0.020	0.807 (0.645–1.010)	0.061
*Marital status*		**0.031**		0.146
Married	Reference		Reference	
Single	1.523 (1.110–2.089)	0.09	1.367 (0.973–1.922)	0.072
S/D/W	1.144 (0.914–1.431)	0.240	1.180 (0.928–1.501)	0.177
*Summary stage*		**< 0.001**		**< 0.001**
Localized	Reference		Reference	
Regional	1.657 (1.174–2.340)	0.004	1.764 (1.239–2.514)	0.002
Distant	6.247 (4.430–8.809)	< 0.001	4.223 (2.755–6.473)	**< **0.001
*Laterality*		**< 0.001**		0.288
Left	Reference		Reference	
Right	0.953 (0.770–1.178)	0.656	1.090 (0.872–1.362)	0.450
Bilateral	2.625(1.696–4.063)	< 0.001	0.726 (0.432–1.220)	0.226
*Primary site*		**< 0.001**		0.074
Upper lobe	Reference		Reference	
Main Bronchus	2.410 (1.181–4.915)	0.016	1.443 (0.695–2.998)	0.326
Middle lobe	1.263 (0.778–2.048)	0.345	1.304 (0.785–2.166)	0.305
Lower lobe	1.144 (0.881–1.485)	0.313	1.126 (0.859–1.476)	0.390
Overlapping lesions	2.290 (1.734–3.026)	< 0.001	1.610 (1.150–2.255)	0.006
*Surgery*		**< 0.001**		**< 0.001**
No	Reference		Reference	
Yes	0.253 (0.202–0.316)	< 0.001	0.470 (0.339–0.653)	< 0.001
*Chemotherapy*		**0.045**		**0.002**
No	Reference		Reference	
Yes	0.803 (0.649–0.995)	0.045	0.683 (0.535–0.873)	0.002
*Radiation*		**0.183**		0.604
No	Reference		Reference	
Yes	1.071 (0.799–1.435)	**0.183**	0.938 (0.738–1.193)	0.604

Statistical analyses were performed using SPSS

*S/D/W* : separated/divorced/widowed

Bold means statistically significant value *p* < 0.05

Considering stage determines the treatment, we conducted the subgroup analysis stratified by the summary stage to determine the impact of surgery, radiation therapy or chemotherapy on the prognosis. Multivariate Cox analysis showed that surgery was an independent protective factor and this decreased risk of death by 88% (HR = 0.120, 95% CI 0.038–0.383) and 65% (HR = 0.351, 95% CI 0.212–0.582) for patients in localized stage and regional stage, respectively (Table 3). However, survival could barely benefit from surgery in these patients with distant stage. In localized stage, we were surprised to find patients who received chemotherapy had significantly poorer prognosis than those who did not (*p* = 0.023). And there was no significant survival difference for patients with regional stages between chemotherapy therapy group and non-chemotherapy group. But in distant stage, chemotherapy was an independent protective factor and this decreased risk of death by 46% (HR = 0.544, 95% CI 0.393–0.752) for patients (Table 3). Similarly, we found that there were no significant survival difference for patients with different SEER summary stages between radiation therapy group and non-radiation group in multivariate analysis.

**Table 3:** Multivariate Cox proportional hazard analyses of SEER summary stage for overall survival rates in patients with PPC

	Localized stage		Regional stage		Distant stage	
	HR (95% CI)	*p* value	HR (95% CI)	*p* value	HR (95% CI)	*p* value
*Surgery*		**< 0.001**		**< 0.000**		0.192
No	Reference		Reference		Reference	
Yes	0.120 (0.038–0.383)		0.351 (0.212–0.582)		0.741 (0.472–1.162)	
*Chemotherapy*		**0.023**		0.300		**< 0.001**
No	Reference		Reference		Reference	
Yes	2.781 (1.151–6.720)		0.791 (0.508–1.232)		0.544 (0.393–0.752)	
*Radiation*		0.898		0.380		0.268
No	Reference		Reference		Reference	
Yes	0.930 (0.305–2.833)		0.805 (0.497–1.306)		0.839 (0.615–1.144)	

Statistical analyses were performed using SPSS

Bold means statistically significant value *p* < 0.05

## Discussion

Pulmonary pleomorphic carcinoma (PPC) is a rare malignant tumor that combines spindle or giant cell carcinoma with any of the more common types of non-small-cell lung cancer (NSCLC) [13]. Reviewing the literature, the incidence of PPC has been reported to range from 0.1 to 0.4% of all pulmonary malignancies [8]. Owing to the limitation of biopsy tissues, PPC sometimes may be diagnosed inaccurately and easily misdiagnosed as the other 4 subtypes of sarcomatoid cancer [17]. Some cases have been reported that some atypical symptoms of patients with PPC may appear, such as chest pain, irritate cough, hemosputum, fever and weight loss [18, 19]. Previous studies have shown that PPC commonly occurs in older adults, with a median age of 60–70 years, has a strong male predominance, is associated with smoking [20, 21]. Because of its rarity, except for sporadic case reports and small retrospective case series, there is no adequate data to describe PPC demographics [12]. In our study, PPC is more common in males than in females, with the ratio was about 1.40:1. The mean age of all patients was 66 years and the younger (≤ 66 years) had better OS than older people. Besides, age was to be an independent prognostic factor for PPC. PPC patients were ever reported in different race groups. The exact incidence was not reported in different race groups because of the rare number [17]. However, our analysis demonstrated that about 80.4% of patients with PPC were white people. As the largest analysis of PPC to date, we summarized the correlation between the prognosis of PPC and clinicopathological factors. The 5-year OS of PPC were 23% and the median OS was 9 months, which was consistent with previous literature reports [22, 23]. In addition, our findings indicated that right lung lesions maybe have a better OS than patients with left lung. Lesions were common observed in upper lobe and lesions in lung lobes had a better prognosis compared with those in the main bronchus. Marital status was also a factor affecting survival rate.

Several previous studies, in which large number of patients were analyzed, have reported that TNM stage was associated with poor prognosis [24]. In the present series, TNM stage was also regarded as a prognosis factor of this disease. The TNM stage of PPC was incomplete and only a thirds of them was found in the SEER database. The AJCC stage is a valuable tool for physicians to make treatment plan and prognosis evaluation, which has been widely used in clinical settings [25, 26]. Similarly, the SEER summary stage has simplified and standardized staging to ensure consistent definitions over time, which provide a measure of disease progression [25]. Therefore, we included SEER summary stage instead of TNM stage in univariate analysis and multivariate analysis. In this study, we found that SEER summary stage was associated with OS and the patients with distant stage had significantly poorer OS than those with localized or regional stage. Furthermore, SEER summary stage was independently associated with prognosis in multivariate Cox analysis.

Compared with other subtypes of non-small cell lung cancer, the biological behavior of PPC is highly malignant and even an early tumor may invade the blood vessels, early pulmonary pleomorphic carcinoma without lymphatic metastasis may recur or metastasize. Early detection, early resection, and thorough removal of tumors are important for the treatment of PPC and prevention of recurrence. Surgical treatment only had a certain effect on pulmonary pleomorphic carcinoma without lymph node metastasis, and the operative effect of lymph node metastasis was not obvious [8, 24]. In our study, patients with surgery group had a better OS than non-surgery and the prognosis was significantly improved. Furthermore, we found that surgery could significantly prolong survival in patients with localized stage or regional stage while it did not have great impact on survival in patients with distant stage. Thus, surgery was the first choice for the early stage of pulmonary pleomorphic carcinoma patients.

In terms of adjuvant therapies, PPC as a poor response to chemotherapy and a more progressive clinical course than other types of NSCLC [27], no standard treatments or effective management strategies has been established and there is no consensus on the treatment of PPC even among specialists. Bae et al. [28] reported cytotoxic chemotherapy was administered for postoperative relapse and inoperable cases and 11 of 13 cases involved progressive disease and the median overall survival was about 5 months. Kato et al. resported cisplatin and vinorelbine were effective as a neo-adjuvant therapy for PPC patients [29]. Some reports have mentioned that a regimen involving gemcitabine or taxanes is effective [12, 30, 31]. In our study, we found that patients who received chemotherapy had better OS than patients without chemotherapy and chemotherapy were found to be independently associated with the survival, especially in the distant stage. Besides, chemotherapy therapy did not significantly increase patient survival in regional stage. But in localized stage, chemotherapy could not prolong survival and had significantly poorer prognosis than non-chemotherapy. Review of previous literature, there are few reports about chemotherapy for early PPC, and a number of previous randomized controlled trials have shown that the benefit of adjuvant chemotherapy in early NSCLC is also uncertain [32, 33]. PPC patients received chemotherapy had significantly poorer prognosis than non-chemotherapy in the localized stage, which might be related to the toxicity of chemotherapy drugs. Therefore, we all agree that chemotherapy should be recommended for advanced rather than early pulmonary pleomorphic carcinoma.

Since few studies have reported on the use of radiotherapy for PPC, the therapeutic effect remains unclear [32, 33]. In this study, there was no significant effect on outcomes of radiotherapy. Besides, after adjusting for other variables, no significant survival difference between radiotherapy and non-radiotherapy could be observed in different SEER summary stages. Therefore, the effects of radiotherapy on pulmonary pleomorphic carcinoma need to be further confirmed in multi-center, large-scale clinical studies. In recent years, novel targeted treatments and PD-1 inhibitors have brought new treatment opportunities to advanced patients [34]. Some case reports revealed that Epidermal growth factor receptor (EGFR) mutations or KRAS mutations were identified in some PPC patients [35], satisfactory effects can be obtained in some patients received targeted therapy [36, 37]. Since there were no cases of targeted therapy and immunotherapy in the SEER database, we could not further analyze the data.

Similar to other studies using SEER as a data source, it is acknowledged that our study has limitations. Firstly, as the largest analysis of PPC to date, the sample of our study was still small, due to the low incidence of PPC. However, particularly in the case of the rarer subtypes, it is unlikely that greater case numbers will be found in any other form of study. Secondly, we lack clinical data for most patients, such as SEER summary stage, survival time, treatment and so on. A total of 461 cases were included in the study. Thirdly, a detailed survival analysis of treatment by SEER summary stage was carried out in this study, however the statistical power of this analysis was limited because of low PPC case numbers. Finally, the retrospective nature of our investigation may have introduced bias into the overall analysis.

## Conclusion

The most common type of pleomorphic carcinoma is pulmonary pleomorphic carcinoma. PPC is a rare type of cancer, which has an aggressive clinical course and poor prognosis. The main interest of this study is to predict basic clinicopathologic characteristics and survival. As the largest database available on PPC, a total of 461 PPC patients with complete case information were found in the SEER database. In short, our research indicated that PPC mostly occurred in white people, with a median age of 66 years, has a strong male predominance. Most patients were married and more than half of lesions were observed in the right lung and upper lobe. Moreover, the presence of SEER summary stage, age were to be an independent prognostic factor for PPC, while surgery and chemotherapy was an independent protective factor.

## Data Availability

The original data came from the SEER database. All data discussed in the manuscript are included within this published article.

## Abbreviations

PPC: Pulmonary pleomorphic carcinoma
SEER: Surveillance, epidemiology, and end results
NSCLC: Non-small-cell lung cancer
OS: Overall survival
HR: Hazard ratio
PD-1: Programmed cell death-1
